# Effect of probiotics combined with periodontal basic therapy on inflammatory factors in gingival crevicular fluid in patients with moderate periodontitis

**DOI:** 10.1097/MD.0000000000046471

**Published:** 2025-12-26

**Authors:** Shuo Liu, Tao Song, Jianghui Zhao, Jing Yao

**Affiliations:** a Department of Stomatology, The First Hospital of Handan, Handan, Hebei Province, China.

**Keywords:** basic periodontal treatment, gingival sulcus fluid pro-inflammatory factors, inflammatory factors, moderate periodontitis, periodontal parameters, probiotics

## Abstract

The aim is to evaluate the effect of probiotics combined with basic periodontal treatment on inflammatory factors in gingival crevicular fluid so clarifying the clinical value and mechanism of this combined treatment for moderate periodontitis and so provide evidence for the application of probiotics in its management. From June 2021 to January 2023, a retrospective controlled clinical study was conducted on 140 individuals diagnosed with moderate periodontitis who had received treatment at the Department of Stomatology, the First Hospital of Handan. Based on treatment records, 70 patients who received probiotics combined with standard periodontal basic therapy were assigned to the study group, and 70 patients who received periodontal basic therapy alone were included as the control group. Clinical and laboratory data were collected from patient records, including inflammatory factors (interleukin-6, interleukin-1 beta, high-sensitivity C-reactive protein, tumor necrosis factor-alpha), pro-inflammatory markers (matrix metalloproteinase/tissue inhibitor of metalloproteinase-1, heat shock protein-70, high mobility group box 1), and periodontal parameters (probing depth [PD], GI, PLI, sulcus bleeding index [SBI]), which were measured before and after treatment to evaluate therapeutic effects. PD, GI, PLI, and SBI did not significantly vary across the groups before therapy. After 4 weeks, the study group’s improvements in PD, GI, and PLI were better than those of the control group. At 8 weeks, the study group’s PD, GI, PLI, and SBI were significantly better than those of the control group (*P* < .05). Prior to treatment, there were no changes in matrix metalloproteinase/tissue inhibitor of metalloproteinase-1 or high mobility group box 1, however after 8 weeks, the study group’s levels of pro-inflammatory markers decreased. heat shock protein-70 levels initially differed across the groups, however both showed decreases after treatment, with the study group showing significantly lower levels (*P* < .05). Similarly, there were no early differences in the study group’s levels of interleukin-6, interleukin-1 beta, high-sensitivity C-reactive protein, and tumor necrosis factor-alpha, but after 8 weeks, they considerably dropped (*P* < .05). For individuals with moderate periodontitis, probiotics may significantly reduce inflammatory symptoms when used in conjunction with standard periodontal therapy. Additionally, they have the ability to control the amounts of inflammatory and pro-inflammatory substances. Additionally, they contributed to oral microecology, which is worthwhile to support and advantageous for the management of moderate periodontitis.

## 1. Introduction

The gingiva, periodontal ligament, and alveolar bone are among the supporting tissues of the teeth that are impacted by periodontitis, a chronic inflammatory condition. In varied degrees, it affects 20% to 50% of the world’s population, making it a widespread illness and a serious public health issue. Recent research indicates that periodontitis, especially its severe kind, was classified as the 11th most prevalent illness globally in 2016. Severe periodontitis results in the gradual deterioration of periodontal tissues, particularly the alveolar bone, eventually causing tooth loss, compromised oral function, and diminished quality of life.^[1,2]^ Beyond its immediate impact on dental health, periodontitis is becoming recognized as a significant risk factor for the development and progression of several systemic diseases. These include rheumatoid arthritis, respiratory infections, cardiovascular and cerebrovascular disorders, and diabetes mellitus.^[3–5]^ Individuals impacted by periodontitis have a dual burden, exacerbating both conditions and complicating their management. The pathogenesis of periodontitis begins with microbial infection, and a significant contributing component to this process is the accumulation of pathogenic bacteria in dental plaque. This process begins with the formation of a biofilm, mostly composed of bacteria, on the surface of the tooth.^[6]^ These microorganisms cause gingival inflammation and edema by colonizing the gingival edge and releasing toxins and inflammatory mediators. Over time, the continuous accumulation of plaque, particularly in areas with poor oral hygiene, creates an anaerobic environment that is conducive to the growth of harmful bacteria. These bacteria develop slowly, forming a highly pathogenic biofilm that travels from the supragingival to the subgingival area.^[7]^ As the infection progresses, deeper periodontal tissues become affected, resulting in bone resorption and tissue death. If left untreated, this process accelerates the overall progression of periodontitis, further deteriorating the oral microenvironment while also extending local tissue damage. The etiology of periodontitis is largely influenced by host immunological responses in addition to microbial factors. In response to bacterial invasion, the host immune system produces a number of pro-inflammatory cytokines and enzymes that are intended to eradicate the infection. However, a dysregulated immune response brought on by chronic periodontitis causes an exaggerated inflammatory response that damages the surrounding periodontal tissues in addition to the bacteria.^[8]^ As a result, long-term inflammation exacerbates tissue damage, such as the resorption of alveolar bone and the breakdown of collagen fibers. The progression of periodontitis is made worse by both the direct bacterial attack and immune-mediated tissue destruction. The cornerstone of basic periodontal therapy, the currently recognized treatment for periodontitis, is mechanical debridement of calculus and plaque.^[9]^ This procedure, which is also known as scaling and root planning, aims to remove the biofilm and create a healthy periodontal environment. Although mechanical debridement is effective in reducing gingival inflammation and the number of germs, it is often insufficient, particularly in cases of moderate to severe periodontitis. Consequently, adjuvant therapy, such as the use of antimicrobial medications, are often used to improve therapeutic outcomes.^[10,11]^ The efficiency of antimicrobial therapy is, however, limited by a number of difficulties, including the development of drug-resistant bacterial strains, the potential for adverse systemic reactions, and inadequate drug concentration in the periodontal tissues. These disadvantages highlight the need of additional or different therapy strategies that effectively target the microbial component of periodontitis while reducing systemic side effects. Probiotics have recently become a viable adjuvant in the treatment of periodontal disease. Probiotics are living microorganisms that alter the oral microbiota, promote beneficial bacteria, and suppress detrimental species, thereby providing health benefits to the host. Probiotics are a viable alternative for the treatment of periodontitis in contrast to conventional antimicrobials, as they lack antibiotic resistance and have a favorable safety profile. Furthermore, they possess anti-inflammatory properties that contribute to the mitigation of the excessive inflammatory response associated with periodontitis.^[12]^ Research has shown that probiotics reduce plaque and gingival irritation by inhibiting the development of bacterial biofilms. By modulating the oral microbiota, they aid in the maintenance of oral homeostasis and the slowed progression of periodontal disease. The modulation of pro-inflammatory cytokines in gingival crevicular fluid (GCF) by probiotics prevents alveolar bone resorption, which is a sign of advanced periodontitis.^[13]^ Alterations in GCF inflammatory markers, such as matrix metalloproteinases (MMPs), tissue inhibitors of metalloproteinases (TIMPs), and cytokines, are indicative of the effectiveness of periodontal therapy and reflect the host’s immunological response.^[14]^ The majority of clinical trials focus on oral hygiene metrics, such as plaque and gingival indices, despite the increasing support for the use of probiotics in periodontal treatment. Additionally, there is a scarcity of research that investigates the impact of probiotics on GCF inflammatory markers when used in conjunction with conventional periodontal therapy. Understanding this is essential for the improvement of periodontitis treatment methods and the clarification of the therapeutic mechanisms of probiotics. In order to address this lacuna, this study evaluated the clinical efficacy of probiotics in conjunction with basic periodontal care for moderate cases of periodontitis. It specifically assessed the impact of probiotics on GCF inflammatory and pro-inflammatory variables to elucidate the therapeutic function of probiotics in periodontal disease. One hundred forty patients with moderate periodontitis were randomly assigned to either the experimental group, which received probiotics in conjunction with basic treatment, or the control group, which received basic therapy alone. In order to provide evidence-based insights into the benefits of probiotics in conjunction with routine periodontal care, the levels of important inflammatory markers, such as matrix metalloproteinase-8 (MMP-8), tissue inhibitor of metalloproteinase-1 [TIMP-1], IL-6, interleukin-1 beta (IL-1β), high-sensitivity C-reactive protein (hs-CRP), and TNF-α, were assessed before and after treatment in both groups. In conclusion, this study examines the efficacy of probiotics as a supplement in the treatment of moderate periodontitis. The analysis of the combined impact of probiotics on the GCF inflammatory response in the work contributes to the body of data that demonstrates the benefits of probiotics in dental care. Additionally, it establishes a foundation for future research that is designed to enhance periodontal therapy. By developing a novel, evidence-based approach to periodontitis management, the findings may enhance patient outcomes and enhance the efficacy of periodontal treatment.

## 2. Materials and methods

### 2.1. General information

This study was approved by the Ethics Committee of the First Hospital of Handan. A total of 140 patients with moderate periodontitis who had received treatment at the Department of Stomatology between June 2021 and January 2023 were retrospectively analyzed. According to their treatment modalities, patients were divided into 2 groups: 70 patients who received probiotics combined with basic periodontal therapy were assigned to the study group, and 70 patients who received basic periodontal therapy alone were assigned to the control group.

The sample size estimation was based on previous clinical data, in which the estimated effective rate of conventional therapy was 63%, and that of combination therapy was expected to reach 87%.^[15,16]^ The number of cases in each group = (63% × 37% + 87% × 13%)/(87% − 63%)2 × 10.5 (α = 0.05, β = 0.10) = 63.11. This is considering the 5% to 7% loss-of-visit rate. The sample size of each group was determined to be 70 cases (Table 1).

**Table 1:** Comparison of general information of the 2 groups of patients.

Group	Age (yr*, x* ± *s*)	Gender (n)		Course of disease (yr, *x* ± *s*)	Smoking history (n,%)	Drinking history (n,%)
		Male	Female			
Observation group (n = 70)	60.46 ± 6.94	34	36	2.33 ± 0.81	22 (31.43)	30 (42.86)
Control group (n = 70)	59.33 ± 7.36	37	33	2.39 ± 0.73	19 (27.14)	34 (48.57)
*t*/*x*^2^ value	1.842	0.257		−1.270	0.310	0.461
*P* value	.070	.612		.208	.577	.497

### 2.2. Method

#### 2.2.1. Treatment method

Periodontal fundamental therapy was administered to the control group. This encompassed root planning, supragingival cleansing, and subgingival debridement. Week 1: A full-mouth ultrasonic supragingival cleansing was conducted. Additionally, a hydrogen peroxide rinsing was administered. Additionally, the hemorrhaging was halted and the blood was blow-dried. In the second week of treatment, a full-mouth ultrasonic subgingival debridement was conducted. Subsequently, the treatment was identical to that of week 1. In the third week of treatment, ultrasonic therapy was implemented in conjunction with manual subgingival debridement and root planning. The treatment was then administered in the same manner as in week 1. A full-mouth periodontal examination will be conducted during treatment week 4. If the root surface is smooth and there is no supragingival and subgingival calculus, the surface will be polished. In the event that a site does not satisfy the aforementioned criteria, the ultrasonic method should be repeated in conjunction with manual subgingival scouring and root planning. Subsequently, the identical treatment regimen will be implemented as in week 1. The patients in the observation group were not administered any antimicrobial medications prior to or following the treatment for a period of 6 months. Probiotic tablets were administered to the patients in the study group in accordance with the aforementioned treatment regimen. These tablets (Lactobacillus Royale tablets, Nature’s Way Company, 90 tablets per carton) are to be consumed orally once daily, with 1 capsule consumed each time, for a period of 4 weeks. Both groups received treatment from the same physician. Routine oral hygiene maintenance training and supervision were administered to patients throughout the treatment period. They were directed to document their oral hygiene maintenance on a daily basis.

Patient compliance with probiotic therapy was evaluated retrospectively based on medical and nursing records, which included documentation of medication distribution and patient-reported adherence during follow-up visits. Cases with incomplete medication records or unclear adherence information were excluded from the analysis to ensure data reliability.

#### 2.2.2. Gingival sulcus fluid sample collection and preservation

The gingival sulcus fluid was collected prior to treatment, 4 weeks after treatment, and 8 weeks after treatment, respectively. Sterilized instruments and palms were employed to eliminate the gingival plaque from the observation teeth. A Peri paper filter strip (Oraflow Inc., Smithtown) was inserted into the bottom of the labial gingival sulcus or the periodontal fissures and left for 30 seconds. The filter strip was transferred to an EP tube that contained 0.2 mL of PBS after being removed. The tube was then agitated at 3000 r/min for 1 hour. Finally, centrifuge the mixture for 10 minutes and extract the supernatant.

#### 2.2.3. Gingival sulcus fluid pro-inflammatory factor test

Alveolar bone resorption is associated with matrix metalloproteinase-8 (MMP-8)/matrix metalloproteinase inhibitor-1 (TIMP-1. Excessive destruction of the extracellular matrix is induced by bacterial infection, inflammation invasion of the pulp, and an imbalance in the MMP-8 and TIMP-1 ratios of the gingival sulcus fluid. Dental pulp tissue is somewhat damaged by it. Heat shock protein-70 (HSP-70) is a highly conserved protein that has the potential to exacerbate the inflammatory injury to the dental pulp tissue.^[17]^ A member of the late pro-inflammatory factor family, high mobility group protein 1 (HMGB1) has the ability to activate neutrophils and macrophages. This has the potential to exacerbate the inflammatory response and induce immune dysfunction. ELISA was employed to detect the levels of MMP-8, TIMP1, and HMGB1. MMP-8/TIMP1 was computed. MMP-8 and TIMP1 test kits were supplied by Abcam PLC (Abcam Inc., Cambridge). HMGB1 test kits were supplied by ThermoFisher Scientific (USA). HMGB1 detection instruments were supplied by Scientific. The double antibody sandwich method was employed to detect the level of HSP-70. Shenzhen Huisong Technology Co. supplied the detection equipment.

#### 2.2.4. Detection of inflammatory factor levels in gingival sulcus fluid

Abcam PLC (Abcam Inc.) supplied the test kits for the detection of interleukin-6 (IL-6), interleukin-1 beta (IL-1β), hs-CRP, and tumor necrosis factor-alpha (TNF-α) levels using ELISA.

### 2.3. Observation indicators

#### 2.3.1. Indicators of periodontal parameters

Before the treatment, observations and documentation of 2 groups’ periodontal pocket depth (PD), gingival index (GI), plaque index (PLI), sulcus bleeding index (SBI) were noted. They were noted 8 weeks after the therapy and 4 weeks following another.

#### 2.3.2. Gingival sulcus fluid cytokine and inflammatory factor levels

MMP-8, TIMP1, HSP-70, and HMGB1 levels were identified. The MMP-8/TIMP1 ratio was determined in both groups prior to and following the 8-week treatment period. The gingival sulcus fluid was examined for IL-6, IL-1β, hs-CRP, and TNF-α.

### 2.4. Statistical processing

The data analysis was conducted using SPSS 23.0 (IBM SPSS Statistics, IBM Corp., Armonk). The chi-square test was implemented to analyze categorical data, which were represented as percentages. Means ± standard deviations (*x* ± *s*) were employed for data that was normally distributed, with *t*-tests employed for comparisons. Medians were employed to represent data that did not adhere to a standard distribution. The threshold for statistical significance was established at *P* < .05.

## 3. Result

### 3.1. Comparison of periodontal parameters between the 2 groups

Prior to therapy, the 2 groups’ PD, GI, PLI, and SBI did not vary significantly. After 4 weeks, there was no discernible change in SBI between the observation group and the control group, but there was a considerable improvement in PD, GI, and PLI. The observation group’s PD, GI, PLI, and SBI significantly improved after 8 weeks (*P* < .05, Table 2).

**Table 2:** Comparison of periodontal parameters between the 2 groups (*x* ± *s*).

Group	PD (mm)			GI			PLI			SBI		
	Before treatment	4 wk later	8 wk later	Before treatment	4 wk later	8 wk later	Before treatment	4 wk later	8 wk later	Before treatment	4 wk later	8 wk later
Control (n = 70)	5.77 ± 1.33	4.80 ± 1.26	3.57 ± 1.29	2.83 ± 0.38	2.19 ± 0.39	1.61 ± 0.49	2.81 ± 0.39	2.13 ± 0.87	1.19 ± 0.77	3.43 ± 1.06	2.14 ± 0.57	0.91 ± 0.79
Observation group (n = 70)	5.73 ± 1.33	5.21 ± 1.19	4.39 ± 1.40	2.79 ± 0.41	2.24 ± 0.43	2.04 ± 0.49	2.74 ± 0.44	2.33 ± 0.81	1.53 ± 0.58	3.37 ± 1.04	2.21 ± 0.48	1.53 ± 0.58
*T* value	1.000	−2.894	−4.283	1.758	−2.045	−4.887	2.304	−2.769	−3.778	1.425	−0.779	−6.255
*P* value	.321	.005	<.001	.083	.045	<.001	.024	.007	<.001	.159	.439	<.001

GI = gingival index, PD = probing depth, PLI = plaque index, SBI = sulcus bleeding index.

### 3.2. Comparison of gingival sulcus fluid levels of pro-inflammatory factors between the 2 groups of patients

Likewise, before treatment there were no variations in MMP-8/TIMP1 or HMGB1 levels across groups. But after 8 weeks, the observation group showed reduced pro-inflammatory marker levels relative to the control group (*P* < .05). Initial differences were seen for HSP-70; both groups had lower levels posttreatment (*P* < .05, Table 3). The observation group showed particular lower values.

**Table 3:** Comparison of gingival sulcus fluid levels of pro-inflammatory factors between the 2 groups of patients.

Group	MMP-8/TIMP1		HSP-70 (mg/mL)		HMGB1 (ng/mL)	
	Before treatment	8 wk later	Before treatment	8 wk later	Before treatment	8 wk later
Observation group (n = 70)	15.84 ± 3.01	8.46 ± 2.84	22.07 ± 4.19	7.73 ± 2.62	1.36 ± 0.66	0.71 ± 0.74
Control group (n = 70)	15.56 ± 3.03	12.06 ± 3.35	23.09 ± 3.37	17.09 ± 2.29	1.37 ± 0.66	1.06 ± 0.80
*T* value	1.810	−6.714	−3.364	−21.580	−0.168	−3.778
*P* value	.075	<.001	.001	<.001	.867	<.001

HMGB1 = high mobility group box 1, HSP-70 = heat shock protein-70, MMP-8 = matrix metalloproteinase-8, TIMP-1 = tissue inhibitor of metalloproteinase-1.

### 3.3. Comparison of gingival fluid inflammatory factor levels in 2 groups of patients

Before treatment, the levels of IL-6, IL-1β, hs-CRP, and TNF-α in GCF were comparable between the groups. The observation group exhibited a decrease in these markers in comparison to the control group after 8 weeks (*P* < .05, Table 4).

**Table 4:** Comparison of gingival fluid inflammatory factor levels in 2 groups of patients.

Group	IL-6 (pg/mL)		IL-1β (pg/mL)		hs-CRP (ng/mL)		TNF-α (ng/mL)	
	Before treatment	8 wk later	Before treatment	8 wk later	Before treatment	8 wk later	Before treatment	8 wk later
Observation group (n = 70)	5.84 ± 1.35	3.06 ± 1.20	5.47 ± 1.54	2.79 ± 0.41	9.49 ± 2.33	5.53 ± 1.35	5.57 ± 1.36	3.14 ± 1.15
Control group (n = 70)	5.86 ± 1.37	3.67 ± 1.22	5.46 ± 1.35	4.86 ± 1.28	9.61 ± 2.05	7.71 ± 2.63	5.66 ± 1.32	4.44 ± 1.25
*T* value	−0.125	−4.097	0.061	−12.999	−1.136	−6.068	−0.686	−5.706
*P* value	.901	<.001	.952	<.001	.260	<.001	.495	<.001

hs-CRP = high-sensitivity C-reactive protein, IL-1β = interleukin-1 beta, IL-6 = interleukin-6, TNF-α = tumor necrosis factor-alpha.

## 4. Discussion

Periodontitis is acknowledged as the third disease that poses a hazard to human health in the medical field, following cancer and cardiovascular and cerebrovascular disorders. It poses a significant threat to the oral health of individuals.^[18]^ Periodontitis is a chronic inflammation of the periodontal tissues that is induced by local factors. The age of onset is more prevalent after the age of 35. If gingivitis is not treated promptly, it may progress to periodontitis by spreading from the gingiva to the deeper layers of the periodontium, alveolar bone, and dentin. It is simple to overlook in the early stages since there are no overt symptoms. By the time symptoms appear, it may have progressed to moderate periodontitis. Patients with moderate periodontitis should actively look for more effective therapies based on existing ones.

### 4.1. The clinical efficacy of probiotics in the treatment of moderate periodontitis

The equilibrium of the oral microbiota is disrupted by plaque bacteria, which results in an increase in pathogenic bacteria, a critical factor in the development of periodontal disease. Rinsing alone is ineffective for plaque removal due to its adhesion to teeth, and the therapeutic effect of fundamental periodontal treatment varies among patients. Probiotics, as microbial agents, enhance the microecological equilibrium of the host and regulate the oral flora. Probiotics have been shown to be effective in the treatment of periodontal disease when used in conjunction with periodontal therapy in clinical studies.^[5,19]^ After 4 and 8 weeks of probiotic treatment, the observation group’s PD scores decreased from moderate to mild regions, with values of 4.80 ± 1.26 and 3.57 ± 1.29, respectively, in this study. This implies that the oral flora has been enhanced, and there has been a decrease in gingival recession. The observation group’s GI (1.61 ± 0.49) was considerably lower than that of the control group (2.04 ± 0.49) after 8 weeks, suggesting that gingival inflammation had been improved.^[20]^ Moderate plaque was observed at the gingival margin in both groups prior to treatment. PLI scores decreased after 8 weeks, with thinner plaque layers at the gingival margin as a result of probiotic supplementation. Probiotics have a beneficial effect on immune homeostasis by inhibiting hazardous oral flora.^[21]^ The probiotics significantly improved gingival sulcus hemorrhage; the observation group’s SBI decreased from 3.43 ± 1.06 to 0.91 ± 0.79, while the control group’s SBI was 1.53 ± 0.58, indicating that the gingival health and appearance had been restored.

### 4.2. Probiotics for periodontitis reduce levels of pro-inflammatory factors

Inflammatory factors are released by tissue fluid following the advent of periodontitis. The gingival hemorrhage index is positively correlated with MMP-8, which is synthesized by polymorphonuclear leukocytes. TIMP1, an inhibitor of MMP-8, binds to MMP-8 to prevent its activity.^[22]^ Periodontal inflammation is indicated by elevated MMP-8/TIMP1 levels. This study discovered that probiotic treatment decreased MMP-8/TIMP1 levels in the observation group (8.46 ± 2.84) compared to the control group (12.06 ± 3.35). This suggests that probiotics reduce pro-inflammatory factors by reducing MMP-8, with TIMP1 neutralizing excess MMP-8 to alleviate inflammation.^[23]^ HSP-70 is involved in the promotion of inflammation,^[24]^ whereas HMGB1, which is released upon necrosis induced by periodontal bacteria, stimulates inflammatory responses from fibroblasts, thereby exacerbating periodontal inflammation. HMGB1 (0.71 ± 0.74) and HSP-70 (7.73 ± 2.62) levels in the observation group were considerably lower than in the control group after 8 weeks, suggesting a reduced release of pro-inflammatory factors and a reduced periodontal inflammatory response.

### 4.3. Probiotics in periodontitis reduce levels of inflammatory factors

This study examined the variations in inflammatory components in gingival sulcular fluid to elucidate the mechanism by which probiotics treat periodontitis. The pretreatment results indicated that both groups exhibited elevated levels of IL-6, IL-1β, hs-CRP, and TNF-α, which implies that there was substantial inflammation. Although IL-1β may have a local anti-inflammatory effect, IL-6 induces the division of β-lymphocytes and diminishes the proliferation of fibroblasts. Increased levels of IL-6 and IL-1β are indicative of periodontal inflammation. The local inflammation is represented by TNF-α, while the systemic inflammation that increases with the onset and progression of chronic periodontitis is represented by hs-CRP. Following 8 weeks of probiotic medication, the observation group’s levels of IL-6, IL-1β, hs-CRP, and TNF-α were lower than those of the control group. This suggests that probiotics, in conjunction with basic periodontal care, inhibit immunological responses to plaque bacteria. Along with lowering tissue damage, enhancing periodontal health, boosting antibacterial ability, stabilizing bacterial flora, and enhancing oral microecology, this combination is essential for better patient quality of life and significant potential for broader clinical use.^[25]^ Finally, in moderate periodontitis, combining probiotics with basic periodontal therapy significantly lowers inflammatory symptoms. This approach has strong therapeutic potential since it fundamentally regulates oral microecology, prevents hazardous bacterial growth, decreases pro-inflammatory chemicals, and so reduces inflammation.

Several international studies have reported similar findings regarding the adjunctive benefits of probiotics in periodontal therapy. These demonstrated that *Lactobacillus reuteri* supplementation significantly improved clinical parameters such as PD and GI when combined with scaling and root planing, consistent with our results.^[24]^ Likewise, some studies found that probiotics as a complement to nonsurgical periodontal therapy reduced gingival inflammation and modulated cytokine expression.^[25]^ These findings align with the present study, which also observed significant reductions in IL-6, IL-1β, hs-CRP, and TNF-α levels following combined therapy.

However, compared with some European studies reporting more pronounced long-term benefits, our results showed significant short-term improvement within 8 weeks, possibly due to differences in probiotic strains, dosage forms, and follow-up duration. Future multicenter and long-term studies are warranted to further confirm these effects and explore the optimal probiotic regimens for periodontal management.

## 5. Limitations

This study has several limitations that should be acknowledged. First, as a retrospective controlled study with a relatively small sample size from a single institution, potential selection bias cannot be fully excluded. Second, the observation period was short (8 weeks), which limits the ability to evaluate the long-term clinical effects and stability of probiotic therapy. Third, only 1 probiotic strain (Lactobacillus Royale) was used; the effects of other strains or multi-strain formulations were not assessed. Finally, some clinical parameters and inflammatory markers were measured only at fixed time points, which may not fully capture the dynamic changes in periodontal inflammation. Future studies should include larger, multicenter populations, longer follow-up durations, and diverse probiotic combinations to further validate and expand upon these findings.

## Author contributions

**Conceptualization:** Shuo Liu, Tao Song, Jianghui Zhao, Jing Yao.

**Data curation:** Shuo Liu, Tao Song, Jing Yao.

**Formal analysis:** Shuo Liu, Tao Song, Jianghui Zhao, Jing Yao.

**Funding acquisition:** Shuo Liu.

**Investigation:** Shuo Liu.

**Writing – original draft:** Shuo Liu, Tao Song, Jianghui Zhao, Jing Yao.

**Writing – review & editing:** Shuo Liu, Tao Song, Jianghui Zhao, Jing Yao.
